# Supplementary material for: Desertomycin G, a New Antibiotic with Activity against Mycobacterium tuberculosis and Human Breast Tumor Cell Lines Produced by Streptomyces althioticus MSM3, Isolated from the Cantabrian Sea Intertidal Macroalgae Ulva sp
Source: Mar Drugs. 2019 Feb 12;17(2):114. doi: 10.3390/md17020114 (PMC6409695; doi:10.3390/md17020114)
Supplement: Supplementary file 1 [file marinedrugs-17-00114-s001.zip › Supporting info-marinedrugs-437154.pdf]

Desertomycin G, a new antibiotic with activity against *Mycobacterium tuberculosis* and human breast tumor cell lines produced by *Streptomyces althioticus* MSM3, isolated from the Cantabrian Sea intertidal macroalgae *Ulva* sp.

Alfredo F. Braña[1], Aida Sarmiento-Vizcaíno[1], Ignacio Pérez-Victoria[2], Jesús Martín[2], Luis Otero[3], Juan José Palacios[4], Jonathan Fernández[4], Yamina Mohamedi[5], Tania Fontanil[5], Marina Salmón[1], Santiago Cal[5], Fernando Reyes[2*], Luis A. García[6] and Gloria Blanco[1*]

List of supplementary materials

Figure S1. HPLC-UV trace and UV Spectrum of compound **1**.

Figure S2. ESI-TOF Spectrum of compound **1**.

Figure S3. ^{1}H NMR spectrum (CD_3OD, 500 MHz) of compound **1**.

Figure S4. ^{13}C NMR spectrum (CD_3OD, 125 MHz) of compound **1**.

Figure S5. COSY spectrum of compound **1**.

Figure S6. COSY spectrum of compound **1** (Expansion).

Figure S7. HSQC spectrum of compound **1**.

Figure S8. HMBC spectrum of compound **1**.

Figure S9. NOESY spectrum of compound **1**.

Figure S10. NOESY spectrum of compound **1** (Expansion).

Video 1. Time-lapsed recording of MCF-7 cells (control).

Video 2: Time-lapsed recording of MCF-7 cells treated with 50 mM desertomycin G

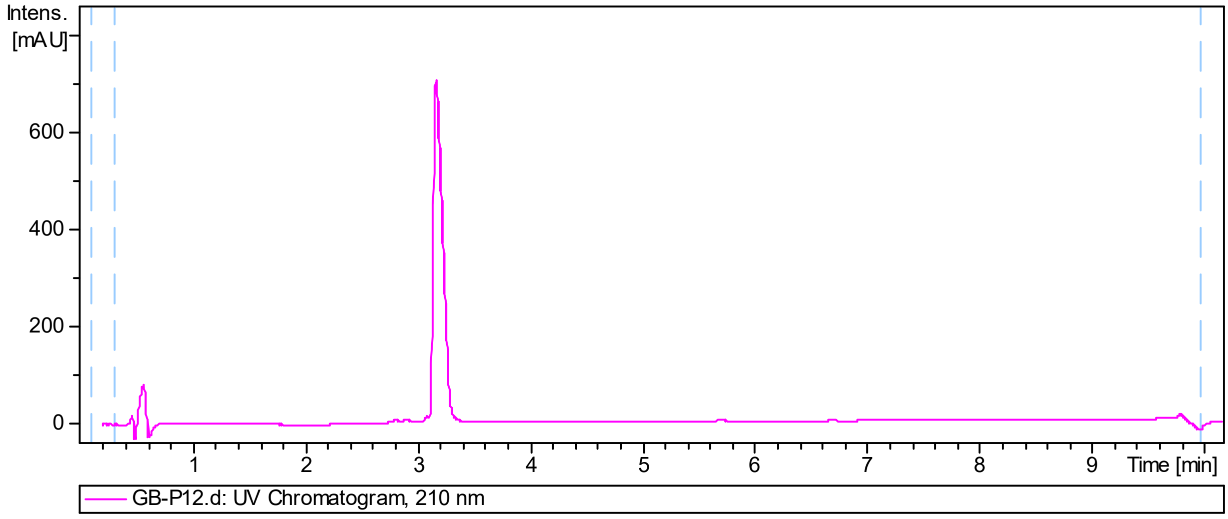

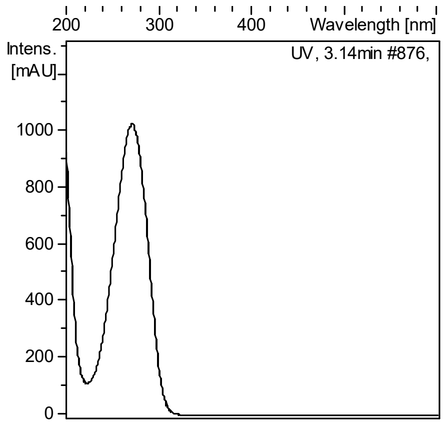

λ_{max}=271nm

Figure S1. HPLC trace and UV spectrum of compound **1**.

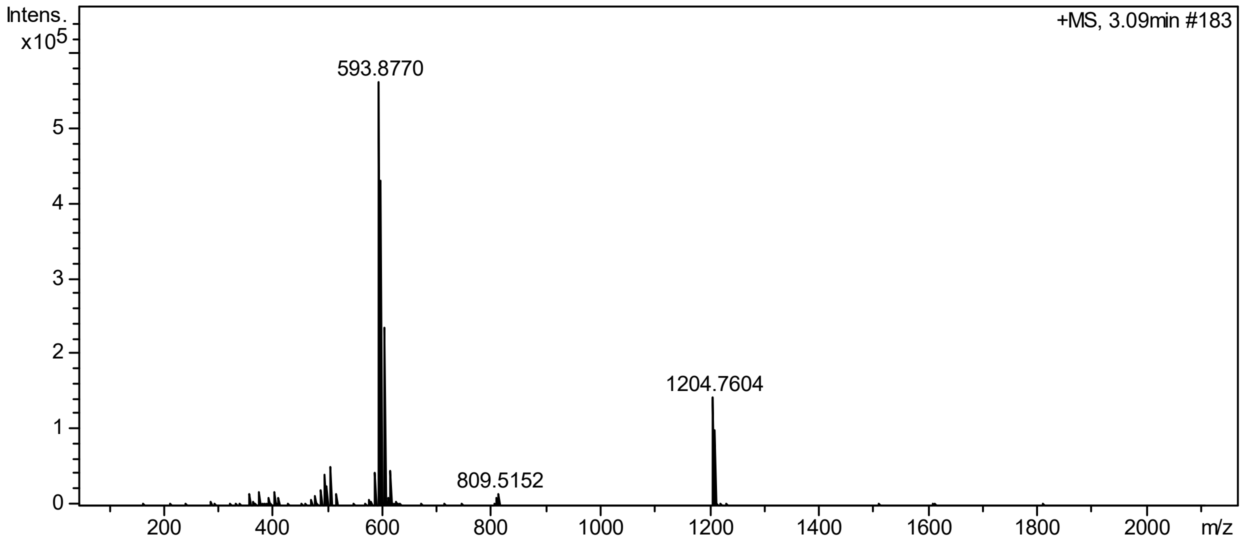

M+H$^+$: $C_{62}H_{110}NO_{21}^+$: 1204.7565

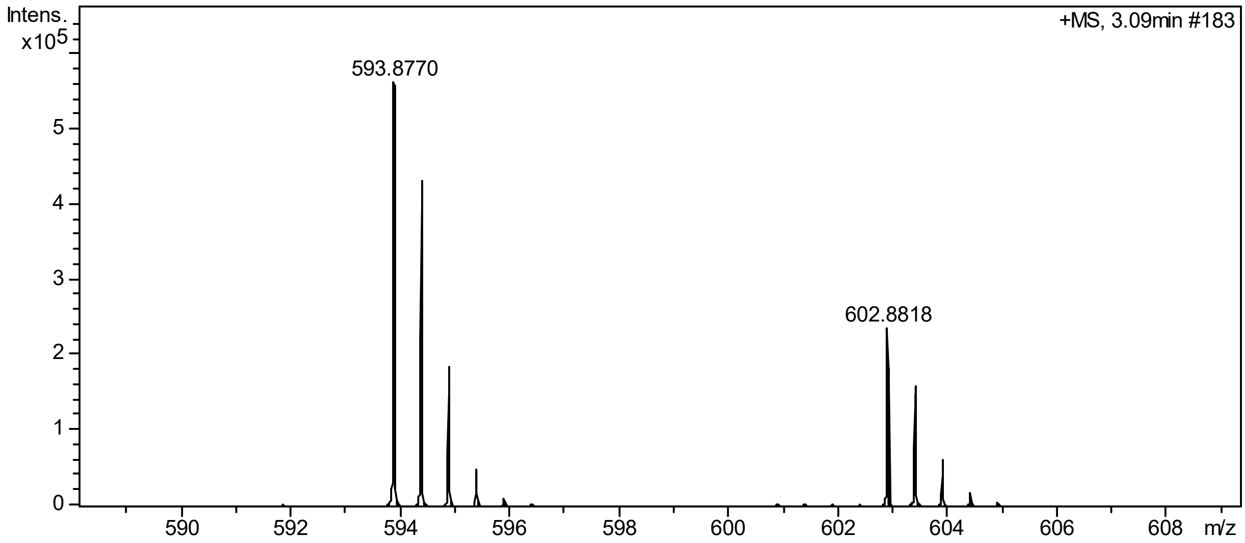

M+2H$^+$: $C_{62}H_{111}NO_{21}^{2+}$: 602.8819
M-H_2O+2H$^+$: $C_{62}H_{109}NO_{20}^{2+}$: 593.8766

Figure S2. ESI-TOF spectrum of compound **1**.

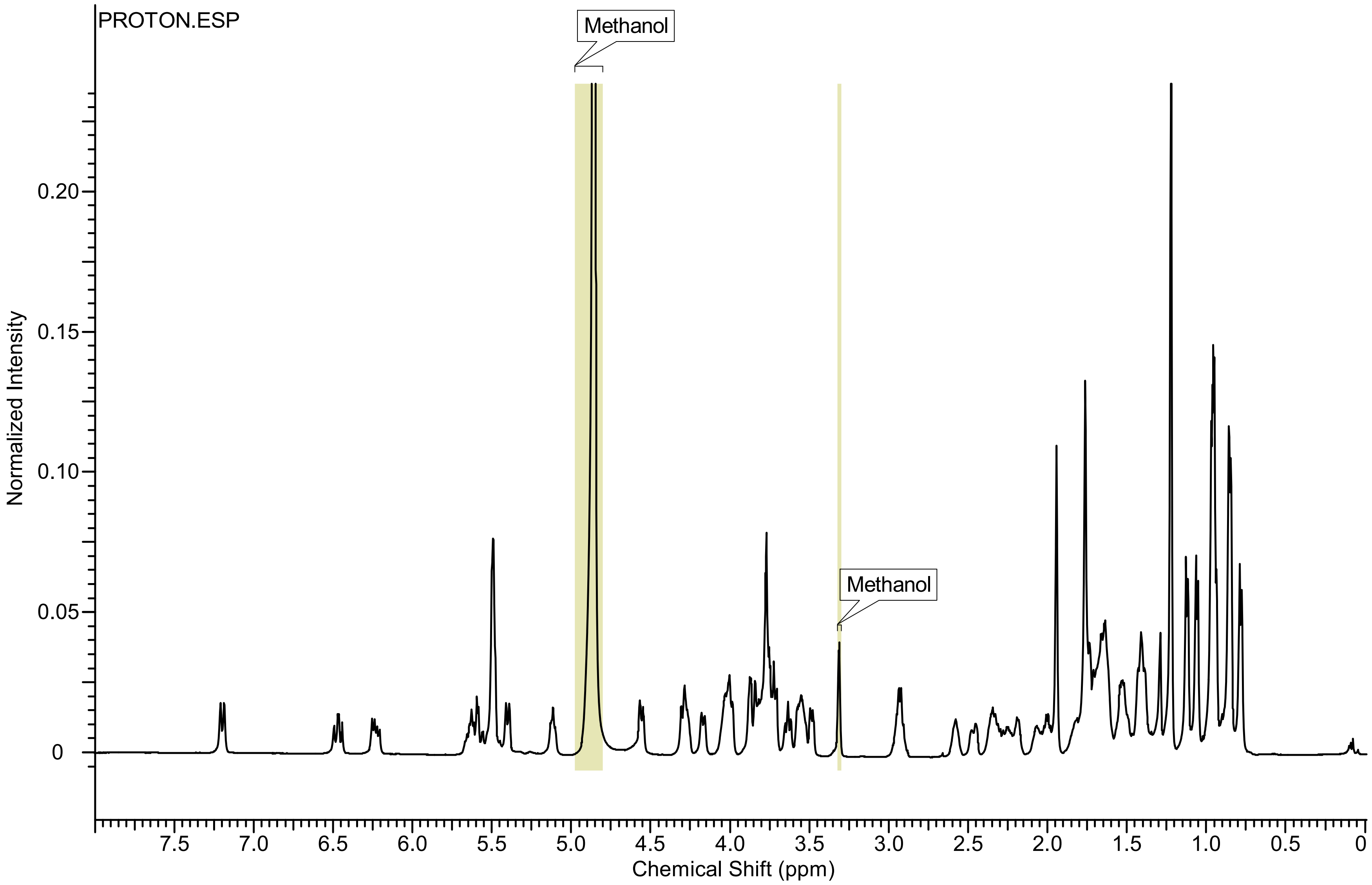

Figure S3. ^{1}H NMR (CD_3OD, 500 MHz) of compound **1**.

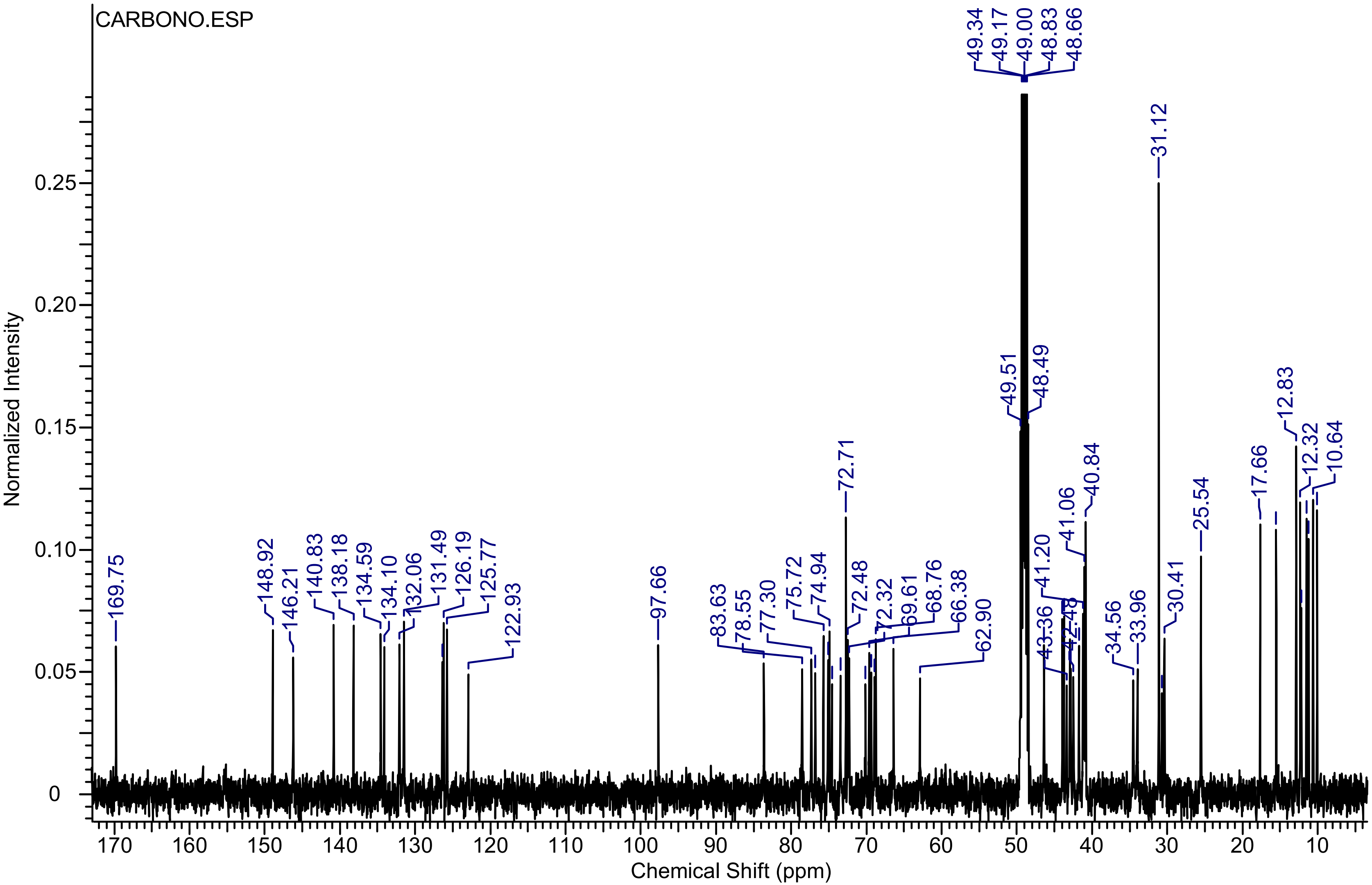

Figure S4. ^{13}C NMR (CD_3OD, 125 MHz) of compound **1**.

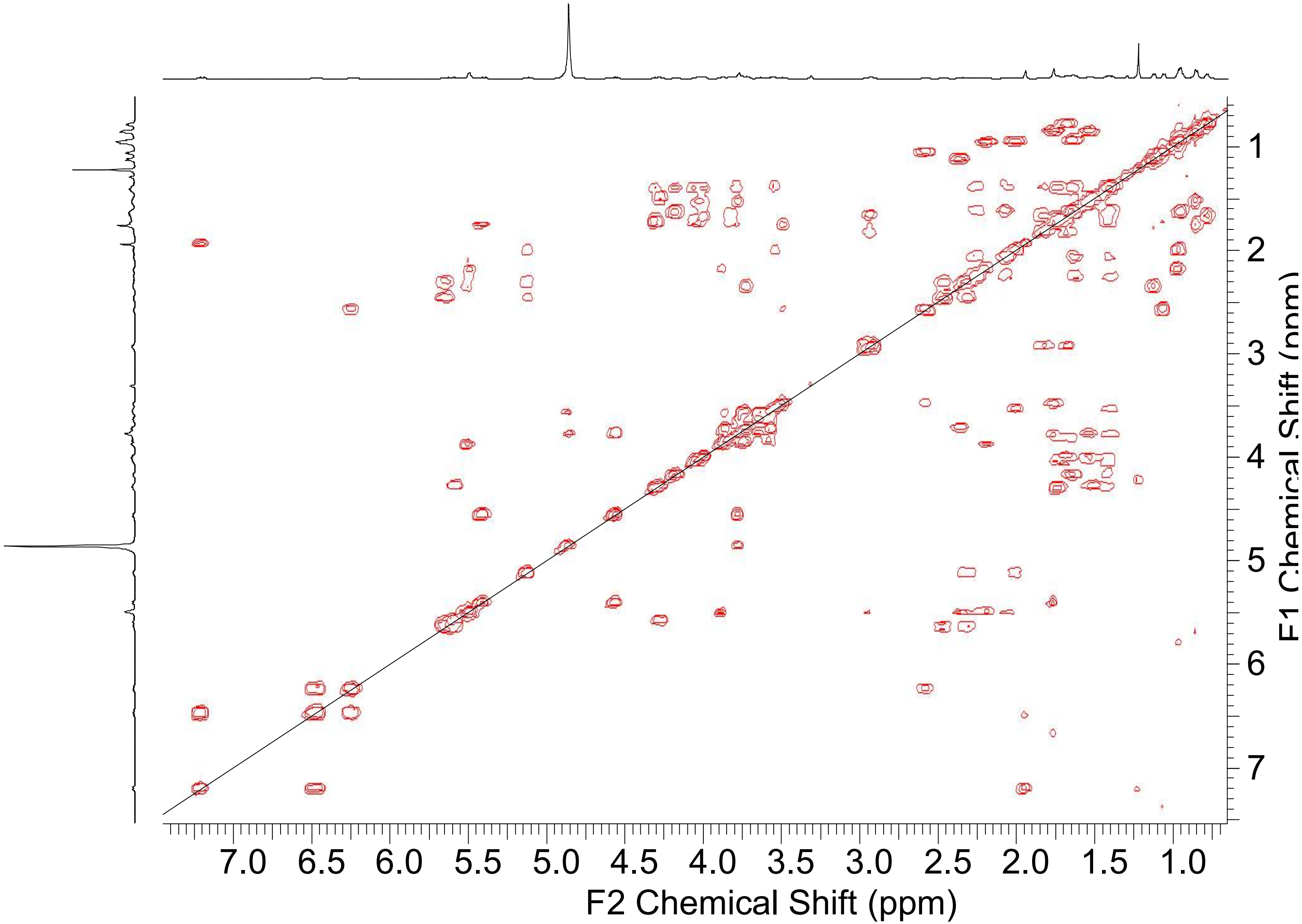

Figure S5. COSY spectrum of compound **1**.

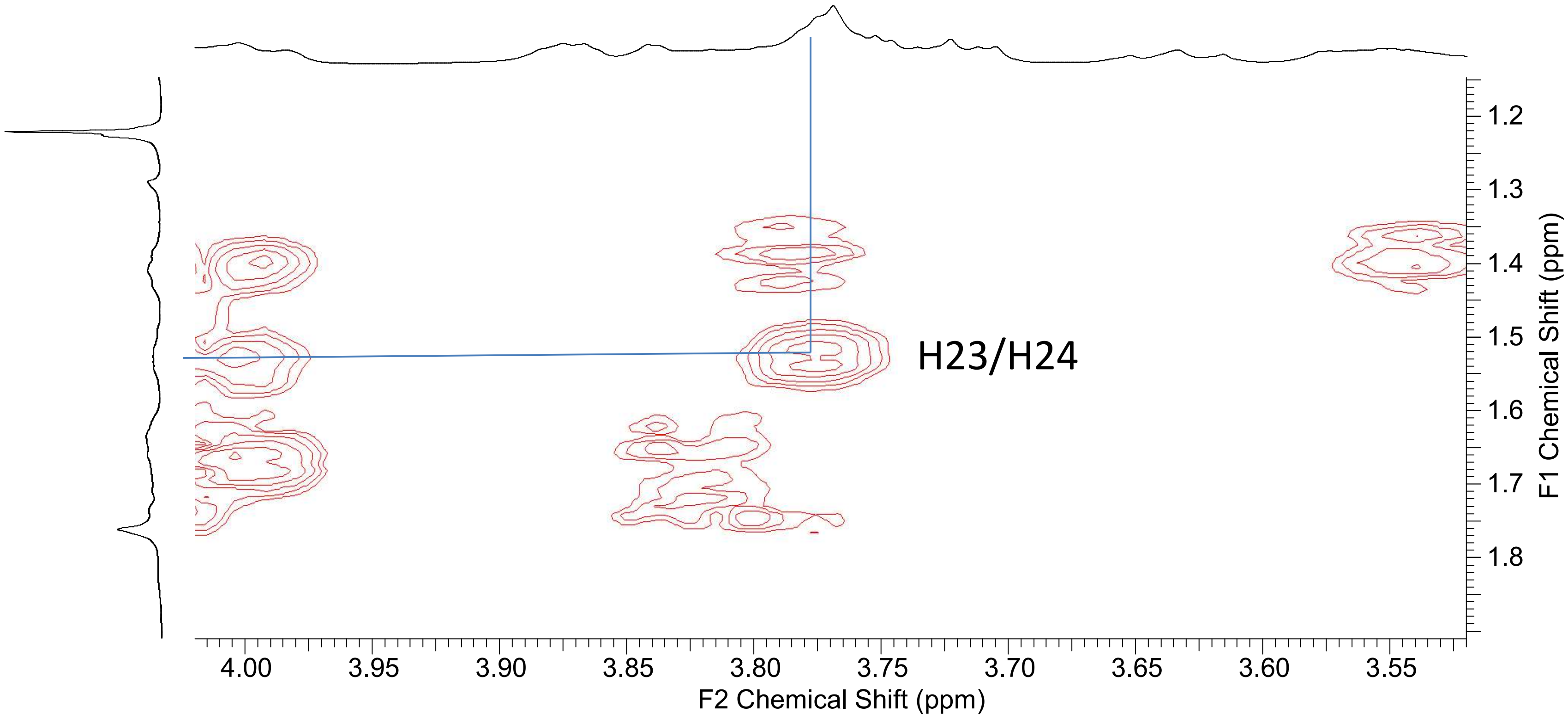

Figure S6. COSY spectrum of compound **1** (expansion).

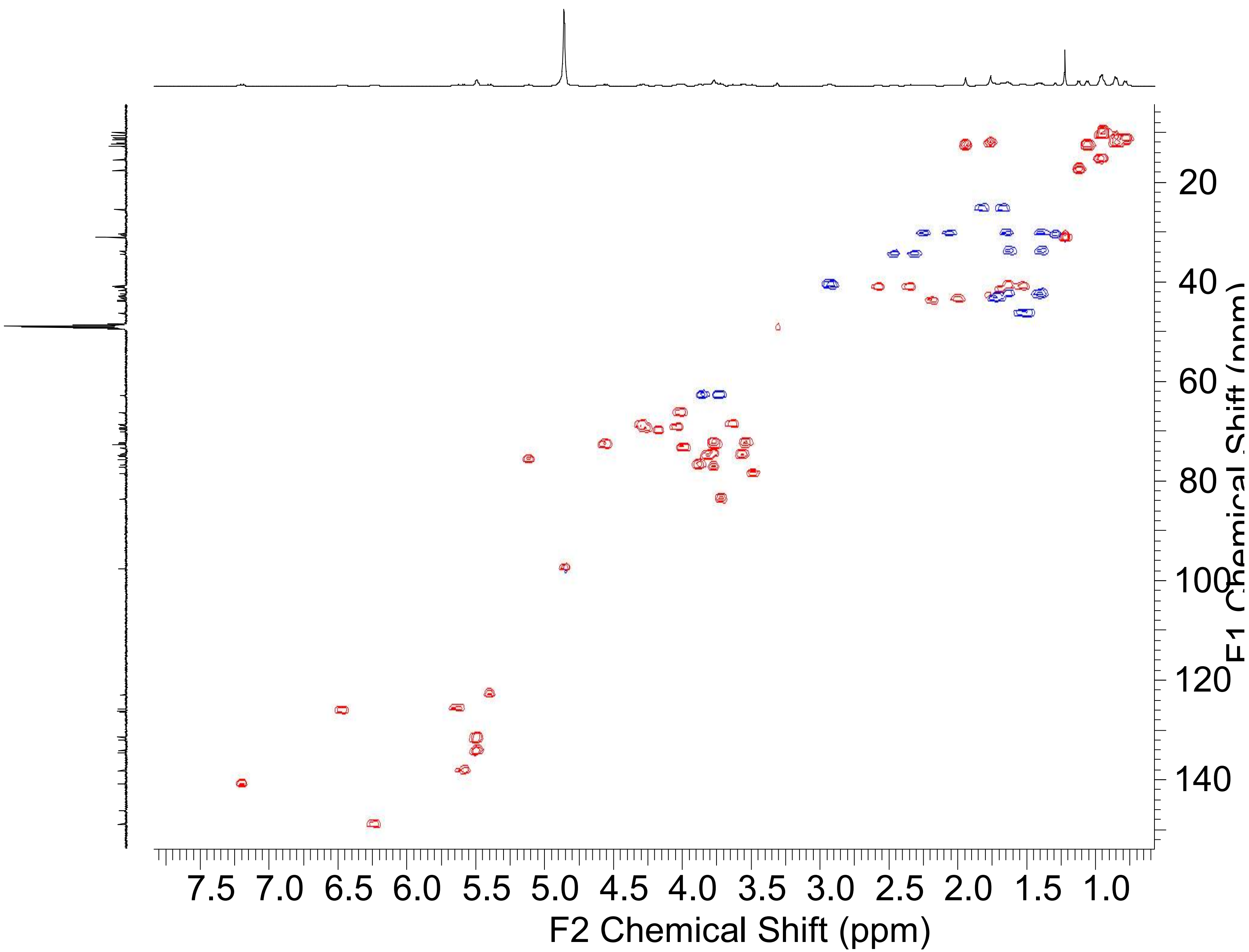

Figure S7. HSQC spectrum of compound **1**.

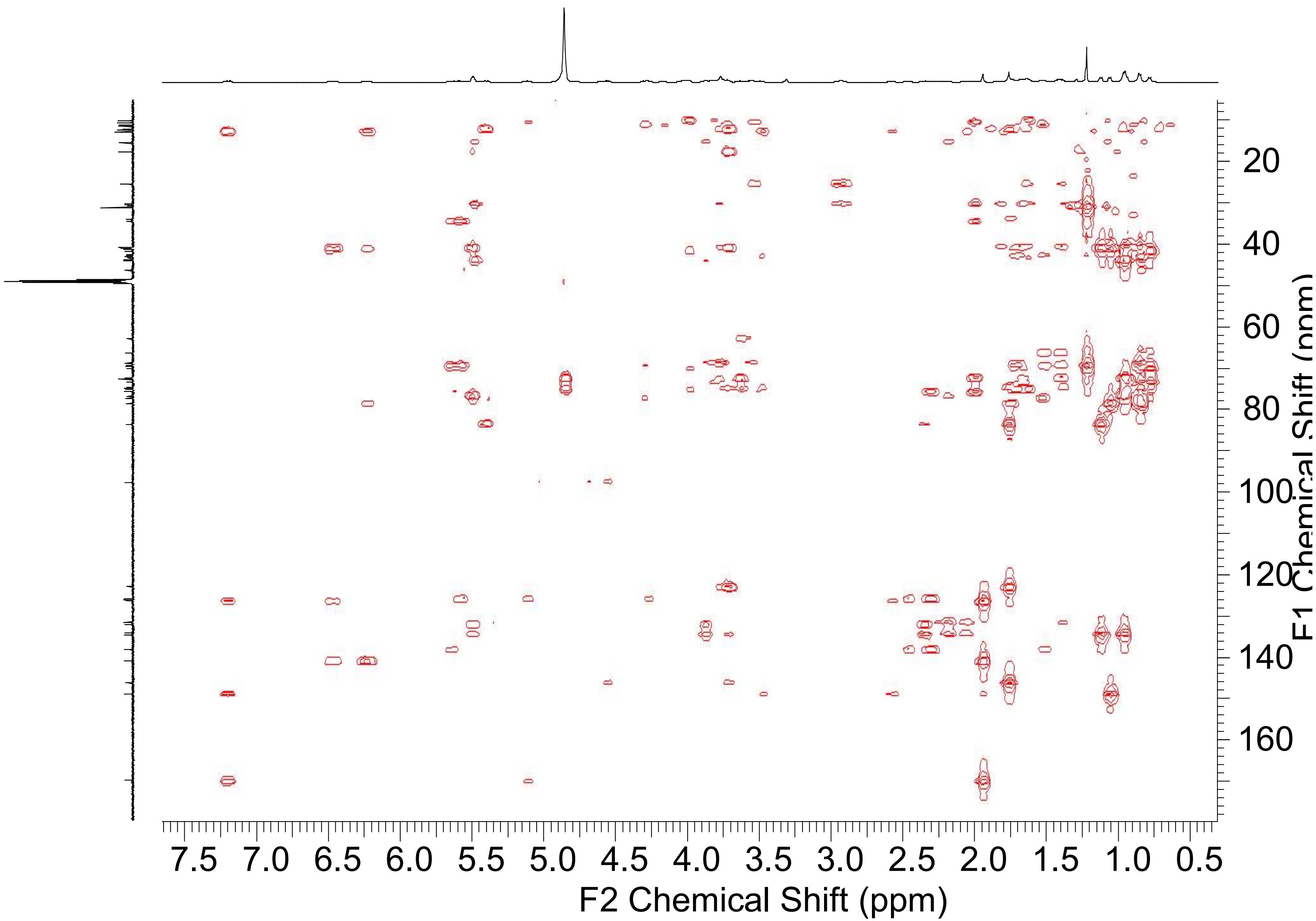

Figure S8. HMBC spectrum of compound **1**.

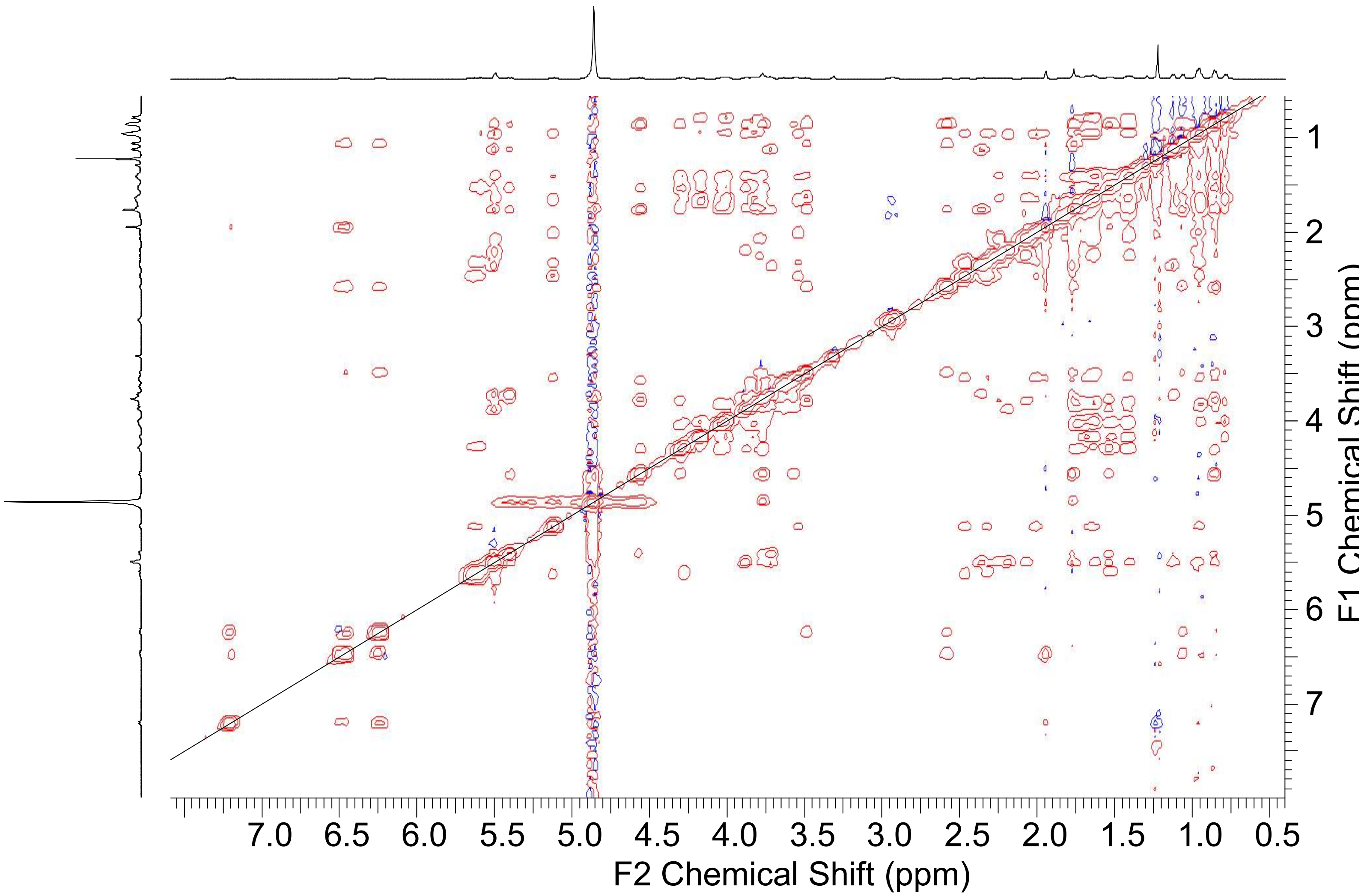

Figure S9. NOESY spectrum of compound **1**.

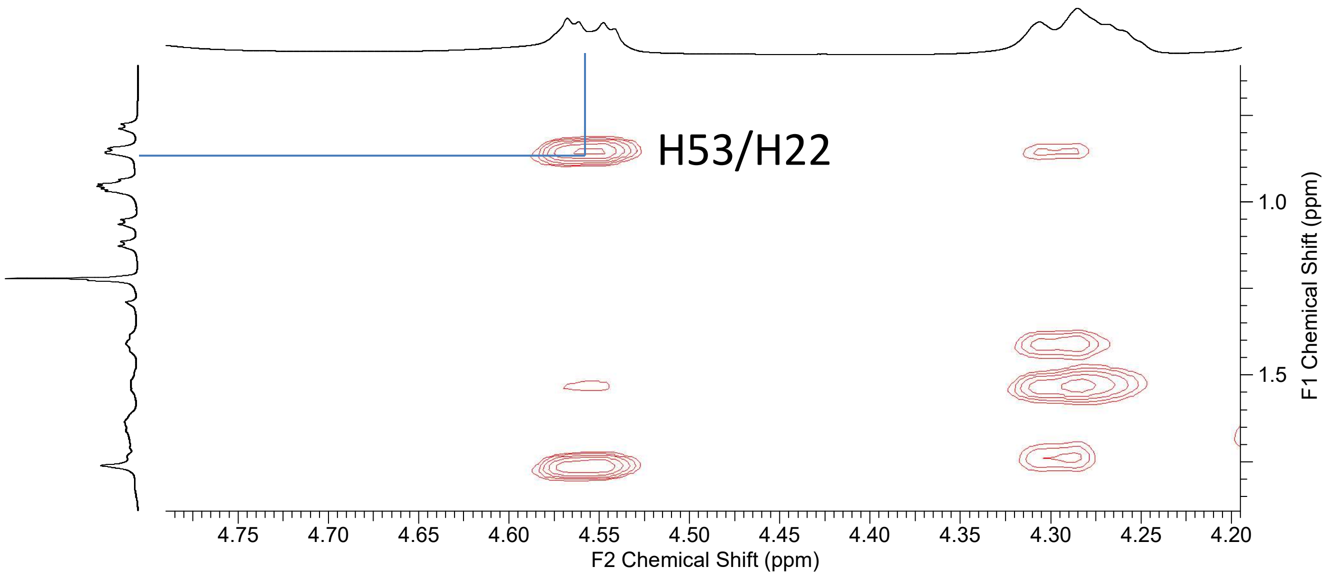

Figure S10. NOESY spectrum of compound **1 (**expansion)
